# Co‐delivery CPT and PTX prodrug with a photo/thermo‐responsive nanoplatform for triple‐negative breast cancer therapy

**DOI:** 10.1002/SMMD.20220036

**Published:** 2022-12-27

**Authors:** Wenhui Zhou, Xiaodong Ma, Jie Wang, Xiaoyu Xu, Oliver Koivisto, Jing Feng, Tapani Viitala, Hongbo Zhang

**Affiliations:** ^1^ Pharmaceutical Sciences Laboratory Åbo Akademi University Turku Finland; ^2^ Turku Bioscience Centre University of Turku and Åbo Akademi University Turku Finland; ^3^ Southern Medical University Affiliated Fengxian Hospital Shanghai China; ^4^ Longgang District People's Hospital of Shenzhen Shenzhen China

**Keywords:** combination chemotherapy, MSN, photothermal therapy, prodrug, TNBC

## Abstract

Triple‐negative breast cancer (TNBC) is still the most aggressive cancer in women. Combination chemotherapy holds great potential for cancer therapy; however, the off‐target and side effects of free chemotherapy administration remain a major challenge. In this study, we developed a photo/thermo‐responsive nanoplatform that can be used for TNBC treatment via photothermic therapy in combination with multidrug therapy. By conjugating the chemotherapy drug PTX prodrug on the surface of mesoporous silica‐coated gold nanorod nanoparticles and then loading another chemotherapy drug, CPT, the Au@MSN‐PTX@CPT nanoparticles exhibited great photothermal response, redox response drug release and cancer cell inhibition abilities. Otherwise, we further coated the Au@MSN‐PTX@CPT nanoparticle with a temperature‐sensitive polymer poly(N‐isopropylacrylamide‐co‐methacrylic acid) (p(NIPAM‐co‐MAAc)), and the polymer‐coated Au@MSN‐PTX@TPT@polymer nanoparticles showed perfect near‐infrared (NIR) light controlled drug release. Finally, the Au@MSN‐PTX@CPT@polymer nanoparticles were injected into the 4T1 breast cancer mouse model. The Au@MSN‐PTX@CPT@polymer nanoparticles preferably accumulated at the tumor site and had reduced chemotherapy injuries and great antitumor activity when combined with 650 nm laser treatment. In summary, our developed Au@MSN‐PTX@CPT@polymer nanoparticles served as a good method for controlled chemodrug delivery and provided a good choice for TNBC combination therapy.

## Graphical abstract

The schematic of Au@MSN‐PTX@CPT@polymer NPs preparation and working model.

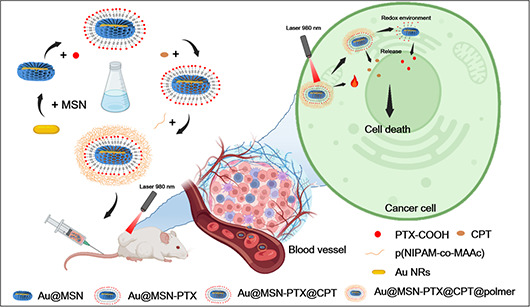

1

**Key points**

- PTX prodrug is synthesized for combination chemotherapy.
- A photo/thermos responsive nanoplatform for TNBC combination therapy is developed.
- Near‐infrared (NIR) light‐controlled drug release is realized.
- The nanoplatform exhibits redox responsive drug release and preferred tumor tissue accumulation.
- It provides a good choice for TNBC treatment via photothermic therapy in combination with multidrug therapy.

## 2 INTRODUCTION

Triple‐negative breast cancer (TNBC) is one of the most aggressive breast cancer subtypes and has high rates of recurrence and metastasis and a poor prognosis.^1^ The main methods for breast cancer treatment include surgery, chemotherapy, hormonal therapy, and targeted drug therapy.^1^ ^b^ However, due to the lack expression of progesterone receptor (PR), estrogen receptor (ER) and human epidermal growth factor receptor 2 (HER2), TNBC does not respond to most developed breast cancer target drugs^1^ ^a,^ ^2^; thus, chemotherapy is still the only option for TNBC patients.^3^ However, the treatment is unsatisfactory because of frequently occurring drug resistance, and the overall survival is only 10–13 months. Therefore, the development of new therapies for the treatment of TNBC is still in high demand.

Combination chemotherapy, the use of two or more chemotherapy drugs for cancer treatment, has emerged as a widely used method for cancer treatment in the clinic because of reduced chemotherapy drug administration and the potential synergistic antitumor activity of chemotherapy drugs.^4^ It was reported that the combined use of multiple chemotherapy drugs reduced drug resistance owing to its multiple antitumor activities. Targeting different cell pathways by the combination of several different chemotherapy drugs enhanced the antitumor effect.^5^ In addition, the cytotoxicity of chemotherapy drugs can be reduced via the selection using of certain chemotherapy drugs. For example, the toxicity of alkylating agents can be reduced by using euclide compounds. Combination chemotherapy is also used for the treatment of breast cancer, for example, the commonly used regimens TAC (the combination of paclitaxel [PTX], doxorubicin [DOX] and cyclophosphamide [CTX]) and AT (the combination of DOX and PTX).^6^ Therefore, the combination therapy holds great potential for TNBC treatment.^7^ However, despite the big advantages of combination therapy, unexpected drug interactions and the special drug duration of action may also cause some irreversible side effects for the hosts. Therefore, the ratio of each combined chemotherapy drug needs to be controlled precisely. Otherwise, the order for chemotherapy drug administration should also be considered.^8^

Prodrugs are compounds that can be catalyzed into pharmacological molecules once they enter the human body.^9^ According to the modification methods, prodrugs can be divided into carrier‐linked prodrugs and bioprecursor prodrugs. Both of these prodrug forms can be regarded as good strategies for improving bioavailability and stability, reducing side effects, and prolonging the release of certain drugs.^10^ For example, by introducing a trisulfide bond to DOX, a redox‐sensitive nanoparticulate drug delivery system for cancer therapy was developed.^11^ Moreover, by using heterotelechelic polymer prodrugs, the loading of both different chemotherapy drug combinations and different chemotherapy drug ratios in the same nanocarrier was realized.^12^ Therefore, prodrug technology is an ideal choice for promoting combination chemotherapy.

In this study, to provide a good solution for combination chemotherapy‐based TNBC treatment, we first conjugated a PTX prodrug on the surface of mesoporous silicon‐coated gold nanorods (Au NRs) via disulfide bonds to form Au@MSN‐PTX nanoparticles (NPs). The redox‐sensitive disulfide bond can be catalyzed by the redox microenvironment of tumor tissue; therefore, PTX will be released to kill cancer cells. Meanwhile, the Au NRs in the core of Au@MSN‐PTX NPs will also be able to generate a photothermal response under 980 nm laser irradiation. Thus, combination chemotherapy and photothermal therapy (PTT) are realized. Furthermore, another chemotherapy drug, camptothecin (CPT), was loaded into the MSN pore structures at the best PTX and CPT mass ratio prior to a temperature‐sensitive polymer poly(N‐isopropylacrylamide‐co‐methacrylic acid) (p(NIPAM‐co‐MAAc)) coating. Finally, the formed Au@MSN‐PTX@CPT@polymer NPs exhibited a perfect photothermal response, redox‐controlled PTX release and enhanced antitumor activity both in vitro and in vivo. In general, our developed Au@MSN‐PTX@CPT@polymer NPs provided a good solution for combination chemotherapy and PTT‐based cancer treatment and offered an ideal nanocarrier for controlled release of chemotherapy drugs.

## 3 RESULTS AND DISCUSSION

### 3.1 PTX‐SS‐COOH synthesis and characterization

**FIGURE 1:**
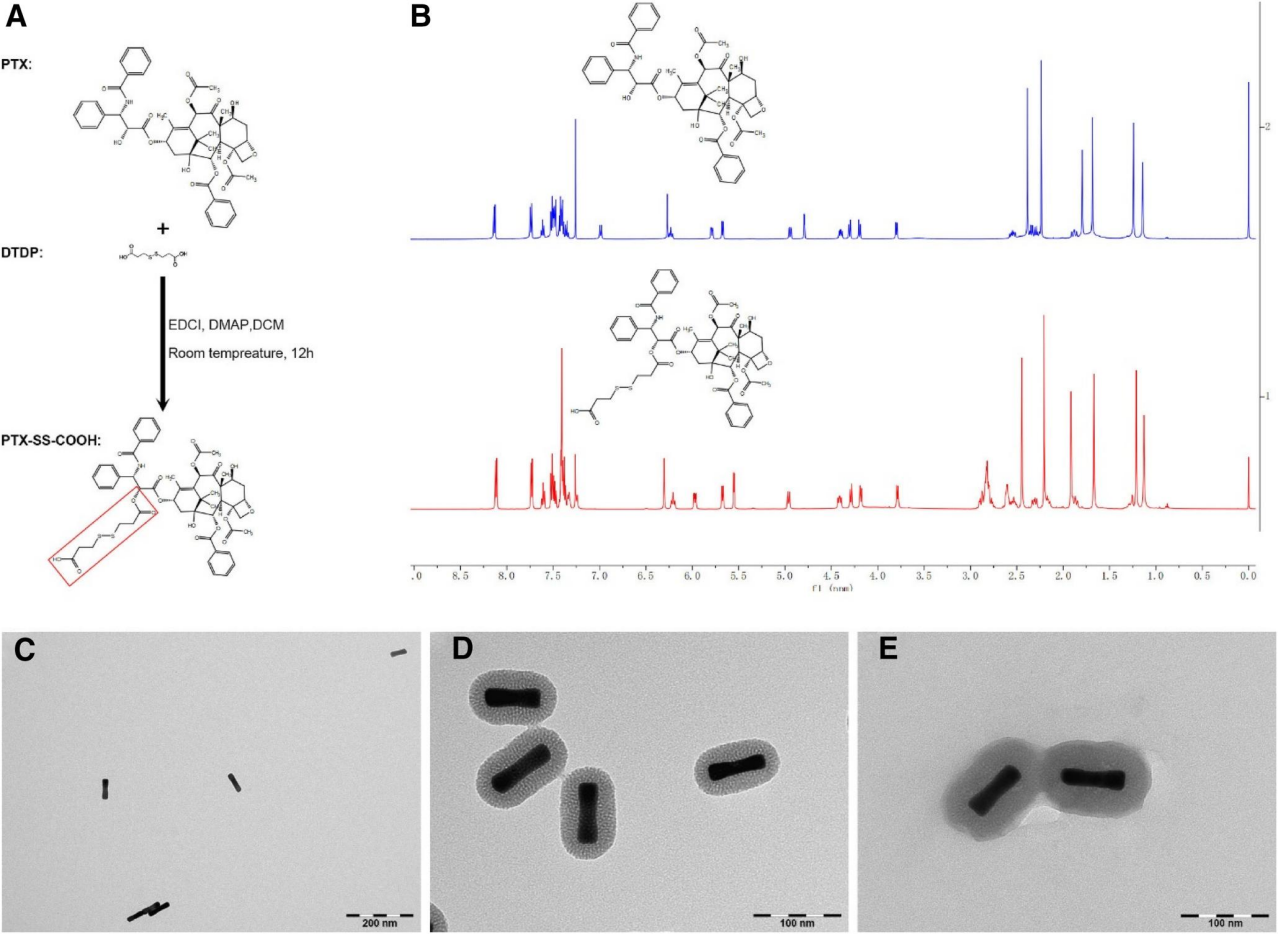
PTX‐SS‐COOH prodrug synthesis and characterization and the morphologies of Au@MSN‐PTX@CPT@polymer NPs. (A) Schematic of PTX‐SS‐COOH synthesis; (B) 1H NMR spectra analysis results; TEM images of Au NRs (C), Au@MSN NPs (D) and Au@MSN‐PTX@CPT@polymer NPs (E).

According to our previous protocol,^13^ the disulfide bond was first introduced to PTX for the prodrug form PTX‐SS‐COOH via a one‐step reaction under a DTDP environment (Figure 1A). As shown in Figure 1B, the 1H NMR spectra analysis result showed new peaks occurred in the range of 2.5–3.0 ppm, which indicated that the redox‐sensitive linker DTDP was successfully linked to the PTX molecule.

### 3.2 Au nanorod and Au@MSN‐PTX@CPT@polymer NPs preparation and characterization

Next, the Au NRs were first prepared by a seed‐mediated growth method in a cetyltrimethylammonium bromide (CTAB) environment as described previously.^14^ After that, the harvested Au NRs were kept in another CTAB environment to generate the MSN layer growth. Subsequently, the PTX‐SS‐COOH and Au@MSN‐NH_2_ NPs were conjugated via activated –COOH and –NH_2_ groups.^15^ Then, the collected Au@MSN‐PTX NPs were coincubated with CPT in DMSO overnight for CPT loading. Finally, the p(NIPAM‐co‐MAAc) polymer was further coated onto the Au@MSN‐PTX@CPT NPs in the water solution to block the release of both PTX and CPT.

**FIGURE 2:**
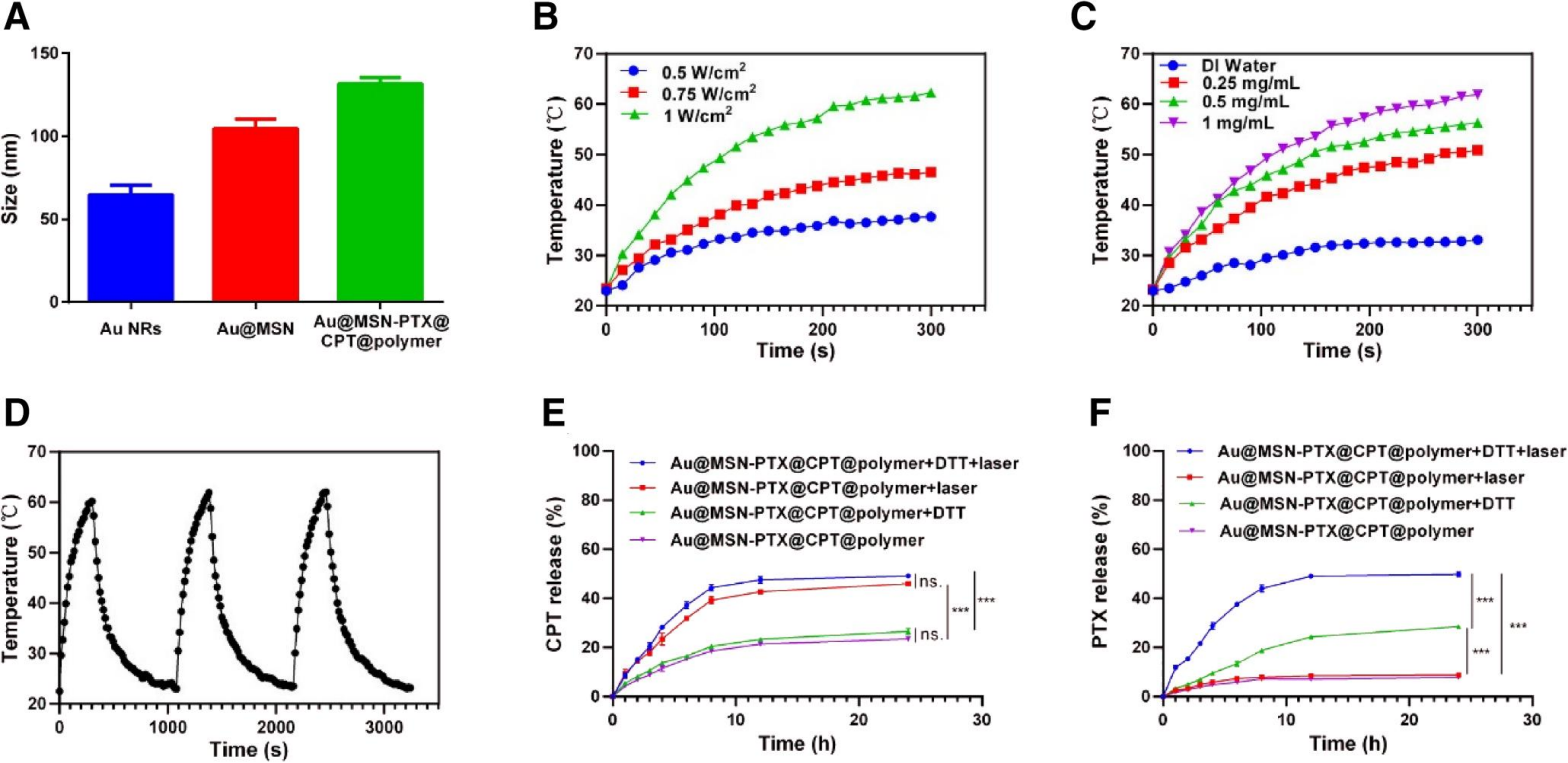
Particle size, photothermal response and drug release of Au@MSN‐PTX@CPT@polymer NPs. (A) Particle size of Au NRs, Au@MSN and Au@MSN‐PTX@CPT@polymer NPs; Photothermal cure of different powers of 980 nm laser treatment (B) and different concentrations of Au@MSN‐PTX@CPT@polymer NPs (C); (D) Photothermal cure of three cycles of NIR on‐off irradiation (1.0 W/cm^2^, 980 nm); CPT (E) and PTX (F) release profiles under DTT (10 mM) and 980 nm laser conditions.

As the result shown in Figures 1C–E, 2A and S1, the particle size of the synthesized Au NRs was 65.7 ± 10.3 nm with a corresponding zeta potential of 9.7 ± 0.7 mV. While after MSN layer growth, a pore structure layer appeared on the surface of the Au NRs, and the particle size increased up to 101.2 ± 15.8 nm. And the zeta potential was increased to 29.5 ± 0.7 mV, which was attributed to the ionizable –NH_2_ groups on the surface of MSN. After that, PTX and CPT loading, as well as the p(NIPAM‐co‐MAAc) polymer coating, made the pore MSN structure of Au@MSN‐PTX@CPT@polymer NPs more compact, but the particle size only increased up to 130.2 ± 25.3 nm. Interestingly, the zeta potential of Au@MSN‐PTX@CPT@polymer NPs was decreased to 14.1 ± 0.7 mV. We believe that this is mainly because PTX‐SS‐COOH interacted with the –NH_2_ groups.

As we hypothesized, the Au NRs can generate a photothermal response, which can be used to combine chemotherapy and PTT, and can also control CPT and PTX release. Therefore, we next performed NIR excitation assays to evaluate the photothermal performance of Au@MSN‐PTX@CPT@polymer NPs. As the result shown in Figure 2B–D, the photothermal conversion by Au@MSN‐PTX@CPT@polymer NPs occurred in a laser power‐ and concentration‐dependent manner. The highest temperature of 64°C was produced by a 1 W/cm^2^ laser and 1 mg/ml Au@MSN‐PTX@CPT@polymer NPs at 300 s. Moreover, a three NIR on‐off irradiation cycle assay also showed that the photothermal conversion ability of Au@MSN‐PTX@CPT@polymer NPs was repeatable and without attenuation. All of these results indicated that the Au@MSN‐PTX@CPT@polymer NPs had perfect photothermal performance. Subsequently, we tested the temperature‐sensitive polymer p(NIPAM‐co‐MAAc) and redox environment controlled drug release. As shown in Figure 2E,F, the CPT release was significantly increased by laser irradiation but not dithiothreitol (DTT). Meanwhile, the PTX release was significantly increased by DTT, while laser irradiation promoted PTX release only under DTT conditions. Since the existence of DTT can best mimic the redox environment, the PTX release only under DTT conditions can be attributed to the disulfide bond breakage under redox catalysis. Overall, these results demonstrated that the Au@MSN‐PTX@CPT@polymer NPs had temperature‐ and redox‐controlled drug releases.

### 3.3 Cell uptake and lysosomal escape of Au@MSN‐PTX@CPT@polymer NPs

**FIGURE 3:**
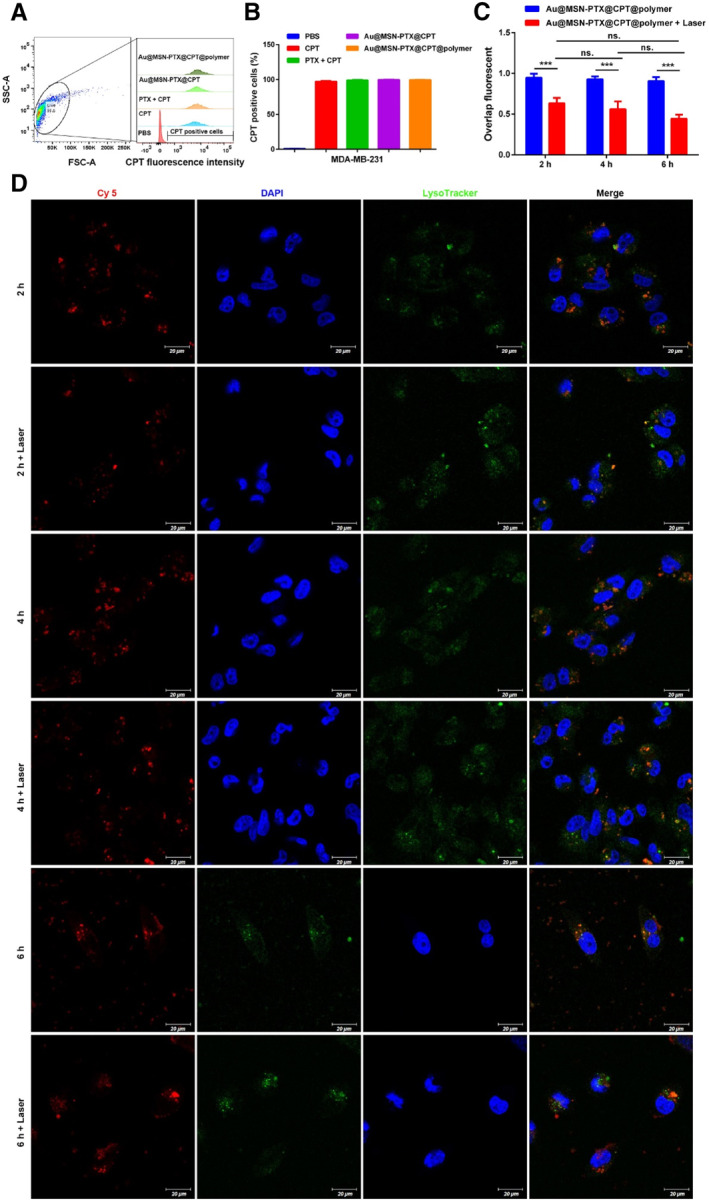
Cell uptake and lysosomal escape of Au@MSN‐PTX@CPT@polymer NPs. Flow cytometry analysis (A) and quantitative results (B) of CPT‐positive cells; Overlap fluorescence (C) and confocal microscopy images (D) of MDA‐MB‐231 cells after incubation with Cy 5‐loaded Au@MSN‐PTX@CPT@polymer NPs for 1–6 h with/without laser irradiation (red: Cy 5; green: Lysotracker; blue: DAPI; scale bar: 20 μm).

Previously, we showed that the Au@MSN‐PTX@CPT@polymer NPs had perfect photothermal performance and controlled drug release. To further investigate whether Au@MSN‐PTX@CPT@polymer NPs can be used for cancer treatment, we further performed cell uptake and lysosomal escape assays to distinguish the interactions between cancer cells and Au@MSN‐PTX@CPT@polymer NPs. Considering that CPT can be excited by 365 nm UV‐light and can emit blue fluorescence at 430 nm, therefore, MDA‐MB‐231 cells after treated with PBS, 10 μg/ml CPT, 10 μg/ml PTX + CPT (mass ratio 1:1), Au@MSN‐PTX@CPT (equal to 10 μg/ml CPT) and Au@MSN‐PTX@CPT@polymer NPs (equal to 10 μg/ml CPT) for 24 h were collected to count the CPT‐positive cells by a flow cytometer. As the result shown in Figure 3A,B, the CPT‐positive cells were all approximately 100% in all of the treated groups compared to PBS groups. The results indicated that Au@MSN‐PTX@CPT and Au@MSN‐PTX@CPT@polymer NPs can freely enter cells as free CPT after 24 h of co‐incubation.

However, the lysosomal escape results showed that (Figure 3C,D) the overlap of Cy 5‐labeled Au@MSN‐PTX@CPT@polymer NPs and LysoTracker was decreased by laser irradiation at all time points, but the overlap decrease followed by incubation time was not significant. The results indicated that laser irradiation is a key promoter of Au@MSN‐PTX@CPT@polymer NPs lysosomal escape.

### 3.4 Antitumor activity of Au@MSN‐PTX@CPT@polymer NPs at the cellular level

To determine whether there are synergistic antitumor effects of CPT and PTX, we treated MDA‐MB‐231 cells in a logarithmic growth phase with a total of 10 μg/ml PTX + CPT (containing 0%–100% PTX). As shown in Figure S4, only 70.07% of MDA‐MB‐231 cells survived after treatment with PTX + CPT (containing 50% PTX) for 48 h, which is the most significant group leading to MDA‐MB‐231 cell growth inhibition. Compared to 94.8% and 72.3% survival rates in the 10 μg/ml CPT‐ and 10 μg/ml PTX‐treated groups, treatment with 10 μg/ml PTX + CPT (containing 50% PTX) obviously enhanced MDA‐MB‐231 inhibition. Therefore, we controlled the loading of CPT into Au@MSN‐PTX@CPT@polymer NPs at a CPT and PTX mass ratio of 1:1 for the final nanoformulation to realize the best antitumor effect.

**FIGURE 4:**
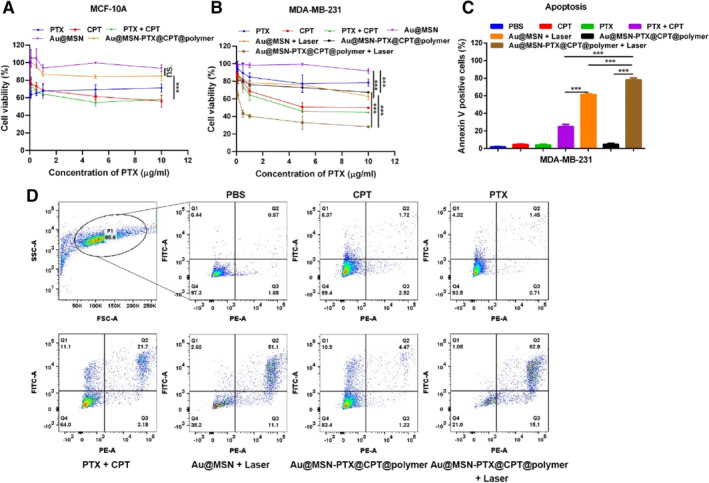
Antitumor activity of Au@MSN‐PTX@CPT@polymer NPs at the cellular level. Cell viability (A, B) and apoptosis analysis (C, D) after treatment with each formulation for 48 h.

Next, we evaluated the antitumor effect on TNBC tissue‐derived MDA‐MB‐231 and normal breast tissue‐derived MCF‐10 cells by WST‐1 cell proliferation assay and Annexin V and PI double staining apoptosis assay. The results in Figure 4A,B show that there was no obvious cytotoxicity of Au@MSN‐PTX@CPT@polymer NPs in MCF‐10A cells. While compared with Au@MSN + Laser and Au@MSN‐PTX@CPT@polymer NPs‐treated groups, the cell viability of Au@MSN‐PTX@CPT@polymer + Laser‐treated MDA‐MB‐231 cells was significantly decreased to 28.3%. And the apoptosis analysis results in Figure 4C,D show that Annexin V‐positive cells were significantly increased in Au@MSN + Laser and Au@MSN‐PTX@CPT@polymer + Laser‐treated groups after 48 h of co‐incubation. However, the increase was not significant in the Au@MSN‐PTX@CPT@polymer NPs‐only treated group. Moreover, the Annexin V‐positive cells in the Au@MSN‐PTX@CPT@polymer + Laser‐treated group was the highest at 78.5%. These results indicated that Au@MSN‐PTX@CPT@polymer + Laser was the most efficient treatment for MDA‐MB‐231 cell inhibition.

### 3.5 In vivo distribution of Au@MSN‐PTX@CPT@polymer NPs

**FIGURE 5:**
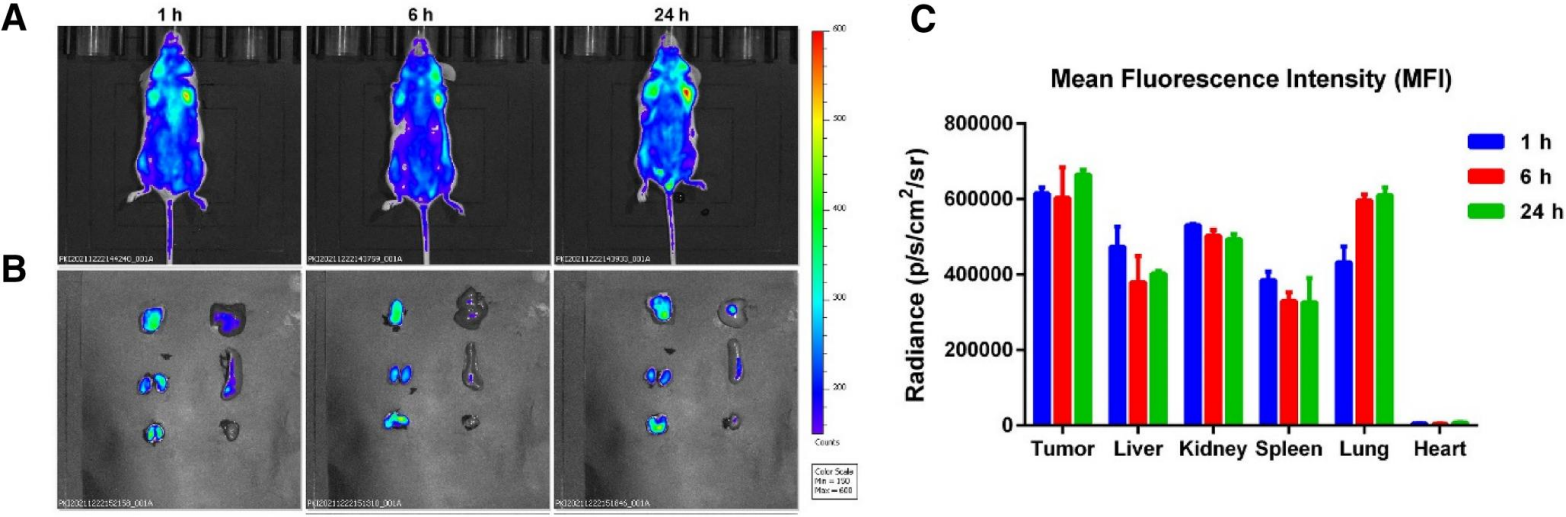
In vivo distribution of Au@MSN‐PTX@CPT@polymer NPs after tail vein injection. Representative fluorescence images of tumor‐bearing mice (A) and major organs (B) after injection with Au@MSN‐PTX@CPT@polymer NPs for 1–24 h from the tail vein (the indicated order of organs in B is tumor, liver, kidney, spleen, lung and heart [from left to right, up to down]); (C) Mean fluorescence intensity of the main organs from each group.

In order to further evaluate the antitumor activity of Au@MSN‐PTX@CPT@polymer NPs in vivo, we first measured the biodistribution in a mouse TNBC tissue‐derived 4T1 cell‐formed xenograft mouse model. After injection with 5 mg/kg Au@MSN‐PTX@CPT@polymer NPs (equal to 5 mg/kg CPT) through the tail vein for interval times, the mice were first anaesthetized for CPT fluorescence signal analysis with an in vivo imaging system (IVIS). After that, the mice were sacrificed for the main organs and tumor tissue imaging. The results in Figure 5A,B show that the Au@MSN‐PTX@CPT@polymer NPs were highly accumulated in tumor tissues only after 1 h of tail vain administration. And the accumulation of Au@MSN‐PTX@CPT@polymer NPs in tumor tissues was gradually increased within 24 h. Interestingly, the amount of Au@MSN‐PTX@CPT@polymer NPs in the liver, kidney and heart was kept at a low level within 24 h. Except for the enhanced permeability and retention (EPR) effect of NPs, this might also be caused by the coated p(NIPAM‐co‐MAAc) polymer, but this still needs to be further verified.

### 3.6 In vivo antitumor activity and cytotoxicity of Au@MSN‐PTX@CPT@polymer NPs

Finally, we evaluated the antitumor activity of Au@MSN‐PTX@CPT@polymer NPs in a 4T1 cell‐formed xenograft mouse model. The treatment was performed by tail vein injection of 5 mg/ml CPT + PTX or Au@MSN‐PTX@CPT@polymer NPs every 3 days, and the next day, the tumor tissue was selectively irradiated by a 1 W/cm^2^ 980 nm laser for 10 min. Tumor volume and mouse body weight were recorded. Once the largest tumor reached to 2 cm in size, the mice were sacrificed to harvest the tumor tissue and main organs. Finally, hematoxylin and eosin (HE) staining was performed to evaluate the cytotoxicity of Au@MSN‐PTX@CPT@polymer NPs in vivo.

**FIGURE 6:**
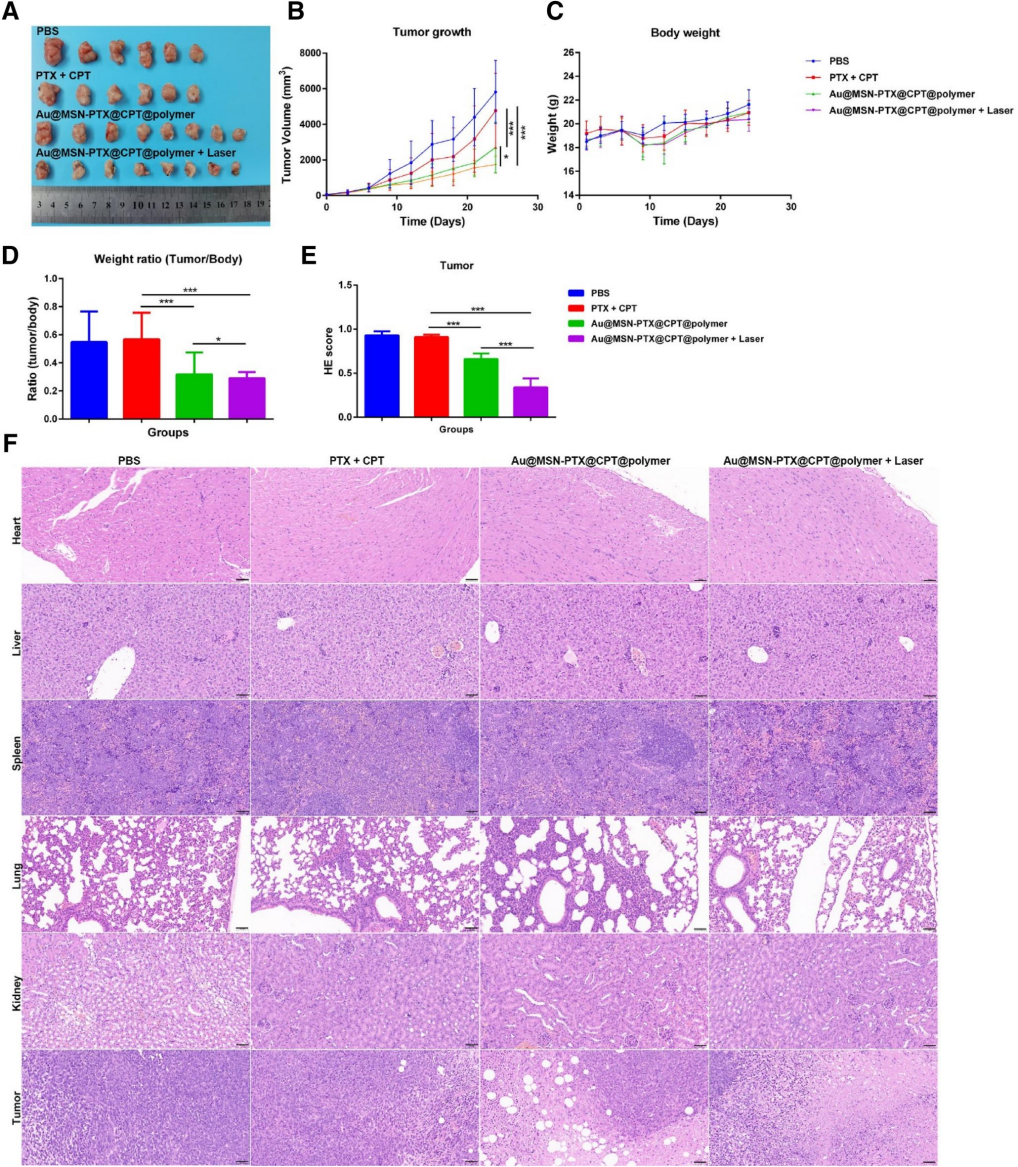
Antitumor activity and cytotoxicity of Au@MSN‐PTX@CPT@polymer NPs in vivo. Tumor tissue (A), tumor growth (B), body weight (C) of mice and tumor/body weight ratio (D) of each formulation treated xenograft mouse model; HE score (E) of tumor tissues and main organs HE staining (F). (Scale bar: 50 μm).

As the results shown in Figure 6A–C, the tumor growth of the 4T1 mouse model was significantly inhibited by Au@MSN‐PTX@CPT@polymer and Au@MSN‐PTX@CPT@polymer + Laser treatment, and the treatment of Au@MSN‐PTX@CPT@polymer + Laser was the most efficient. Moreover, there was no significant body weight change observed during the treatment, and the tumor/body weight ratio results exhibited significantly decreased values in both Au@MSN‐PTX@CPT@polymer and Au@MSN‐PTX@CPT@polymer + Laser‐treated groups (Figure 6D). Furthermore, the HE staining results (Figure 6E,F) showed increased tumor tissue apoptosis in Au@MSN‐PTX@CPT@polymer and Au@MSN‐PTX@CPT@polymer + Laser‐treated groups. Meanwhile, no obvious organ damage occurred in any of the treated groups. All of these results demonstrated that Au@MSN‐PTX@CPT@polymer NPs have good antitumor activity and biocompatibility in vivo, and enhanced antitumor effects can be achieved when combined with 980 nm laser irradiation.

## 4 CONCLUSIONS

In summary, we have developed a redox‐responsive and photothermic controlled CPT and PTX codelivery nanoplatform. The nanoplatform Au@MSN‐PTX@CPT@polymer showed perfect photothermal conversion ability and exhibited great antitumor activity both in vitro and in vivo. Moreover, combination chemotherapy and PTT were realized, and the enhanced antitumor activity was achieved. In general, our developed Au@MSN‐PTX@CPT@polymer NPs provide a controlled chemotherapy drug intracellular delivery nanoplatform and give a good solution for TNBC combination therapy.

## 5 EXPERIMENTAL METHODS

### 5.1 PTX‐SS‐COOH prodrug synthesis

The synthesis of PTX‐SS‐COOH prodrug was generated by an esterification reaction between PTX and 3,3′‐dithiodipropionic acid (DTDP). Briefly, 1.0 g PTX, 295.47 mg DTDP, 171.68 mg 4‐dimethylaminopyridine (DMAP) and 268.51 mg 1‐ethyl‐3‐(3‐dimethylaminopropyl) carbodiimide (EDCI) were robustly dissolved in 20 ml DCM and stirred at room temperature for 12 h. After that, the reaction was stopped, and PTX‐SS‐COOH prodrug was purified by a silica gel column. And the 1H NMR spectra of PTX‐SS‐COOH prodrug was detected.

### 5.2 Au@MSN‐PTX@CPT@polymer NPs preparation and characterization

The Au NRs and Au@MSN‐NH_2_ NPs were prepared by the seed‐mediated growth method as described previously.^13^ After that, Au@MSN‐NH_2_ NPs, PTX‐SS‐COOH prodrug, EDCI and DMAP were dissolved in deionized water and stirred at room temperature for 12 h. Then, the Au@MSN‐PTX NPs were collected and washed three times with deionized water. Next, Au@MSN‐PTX NPs were first stirred with CPT in DMSO for one night and then stirred with p(NIPAM‐co‐MAAc) in deionized water for another night. Finally, the Au@MSN‐PTX@CPT@polymer NPs were collected and characterized by DLS and TEM microscopy.

### 5.3 Drug release assay

Drug release assays were performed with a 10 mM DTT solution. Briefly, 5 mg Au@MSN‐PTX@CPT@polymer NPs were first dissolved in 1 ml of 10 mM DTT solution and then equally divided into 10 1.5 ml EP tubes. At the indicated time points of 1, 2, 3, 4, 6, 8, 12 and 24 h, the Au@MSN‐PTX@CPT@polymer NP dispersed solution was centrifuged at 13,000 rpm for 20 min after irradiation with a 1 W/cm^2^ 980 nm laser for 10 min. Subsequently, 10 μl of supernatant was taken for CPT and PTX measurement by a Nanodrop 2000 (Thermo).

### 5.4 Cell culture

MCF‐10A and MDA‐MB‐231 cells were purchased from American Type Culture Collection (ATCC). MCF‐10A cells were cultured with an MEBM cell culture medium kit. MDA‐MB‐231 cells were cultured in DMEM and supplemented with 10% FBS and 1% PS. And all cells were maintained in an atmosphere of 5% CO_2_ and 37°C.

### 5.5 Cell viability assay

The cell viability was measured with a WST‐1 kit. Briefly, cells were first seeded in to a 96‐well plate at the density of 5000 per well. And next, different amounts of NPs dispersed in the Opti‐MEM medium were added to the cultured cells the next day. Twenty‐four hours later, cells were selectively treated with 1 W/cm^2^ 980 nm laser for 10 min. And 10 μl per well of WST‐1 solution was added to the cultured cells the next day. And after 2 h of further incubation at 37°C, the absorbance (Ab) of each well at 450 nm was measured with a scanning multiwell spectrophotometer. The cell viability was calculated by $Cell\;viability\;\left(\%\right)=\frac{Ab\;value\;\left(experimet\;group\right)}{Ab\;value\;\left(control\;group\right)}\times 100\;\%$. All the experiments were performed in three independent assays, and *p* < 0.05 was considered as significant.

### 5.6 Flow cytometry assay

First, MDA‐MB‐231 cells were seeded in 12‐well plates at a density of 1.5 × 10^5^ per well. The next day, different NP formulations were added to the cells (equal to 10 μg/ml CPT) and incubated for 2–6 h. After that, the cells were washed with PBS and harvested for analysis. And DAPI channel was used for the analysis.

### 5.7 Lysosomal escape assay

Briefly, MDA‐MB‐231 cells were first seeded in 3 cm glass dishes at a density of 3.0 × 10^5^ per dish for 24 h. Next, Au@MSN‐PTX@CPT@polymer NPs labeled with Cy 5 during the synthesis were added to the cells (equal to 10 μg/ml CPT) and incubated for 2–6 h. After that, cells were washed with PBS, and the cell culture medium was replaced with a fresh medium containing 10 mM LysoTracker green dye (Thermo, L7526). Three hours later, the cells were fixed by 4% PFA and stained with 5 μg/ml DAPI. Finally, the cells were imaged by ZEISS LSM880 confocal microscopy.

### 5.8 Animal experiment

All of the animal experiments and operations were conducted by following the guidelines and were approved by theAnimal Research Committee of Shanghai Jiao Tong University School of Medicine, China (SHZY‐202110262). The animal study was performed with 4T1 xenograft mouse models. Briefly, 1 × 10^6^ 4T1 cells in PBS were first injected into the axillary fat pad of 5‐week‐old female nude mice. Once the tumor volume reached to 80 mm^3^, the mice were randomly divided into five groups (six in each group). Among them, one group of the mice was injected with 10 mg/kg Au@MSN‐PTX@CPT@polymer NPs (equal to 10 mg/ml CPT) through the tail vein, and the in vivo distribution of Au@MSN‐PTX@CPT@polymer NPs at 1, 6 and 24 h was detected by an IVIS Lumina imaging system (Capiler). Two another groups were administrated with PBS and 5 mg/kg CPT + PTX (ratio of 1:1), and the two remaining groups were all administrated with 5 mg/kg Au@MSN‐PTX@CPT@polymer NPs (equal to 5 mg/ml CPT) through the tail vein every 3 days. The next day, the tumors from one Au@MSN‐PTX@CPT@polymer NPs‐administrated group were treated with a 1 W/cm^2^ 980 nm laser for 10 min. The body weight and tumor growth were recorded. And the mice were sacrificed once the largest tumor diameter reached 2 cm. Finally, tumor tissues and main organs were obtained, and an HE staining assay was performed.

### 5.9 Statistical analysis

Both quantified data and nonquantified data were collected from triplicate independent experiments. The confocal images were quantified using Image J software. The data analysis and graphical work were performed with GraphPad and SPSS 20.0 software. *p* < 0.05 was considered as significant (* represent *p* < 0.05; ** represent *p* < 0.01; *** represent *p* < 0.001).

## AUTHOR CONTRIBUTIONS

Wenhui Zhou: Experiment design, Cell experiment, data analysis, illustration drawing, original draft preparation and manuscript revision. Xiaodong Ma: Materials synthesis, experiment design, Cell experiment, data analysis, manuscript revision. Jie Wang: Animal experiment. Xiaoyu Xu: Experiment design, manuscript revision. Oliver Koivisto: Manuscript revision. Jing Feng: Data analysis, manuscript revision. Tapani Viitala: Manuscript revision. Hongbo Zhang: Experiment design, provided funding for this project, manuscript revision.

## CONFLICT OF INTEREST

The authors declare no conflict of interest. Hongbo Zhang is a member of the *Smart Medicine* editorial board.

## ETHICS STATEMENT

The project of use and care of the animals in this research “**Co‐delivery CPT and PTX prodrug with a photo/thermo‐responsive nanoplatform for breast cancer therapy**” which was undertaken by Shanghai Jiao Tong University School of Medicine (project leader: Hongbo Zhang) was reviewed and approved by the Institutional Review Board. Animals using in the research were given to the appropriateness of experimental procedures. All animals were lawfully acquired and their retention and use were in every case in compliance with federal, state and local laws and regulations, and in accordance with the Institutional Animal Care and Use Committee of SHZY(IACUC) Guide for Care and Use of Laboratory Animals.

Animals used in this research were received every consideration for their comfort and properly housed, fed, and their surroundings kept in a sanitary condition.

The use of animals was in accordance with the IACUC Guide for Care and Use of Laboratory Animals. The minimal number of mice during the experiment was used in the experiments. Appropriate anesthetics were used to eliminate sensibility to pain during all surgical procedures.

## Supporting information

Supporting Information S1
