# Supplementary material for: Co‐delivery CPT and PTX prodrug with a photo/thermo‐responsive nanoplatform for triple‐negative breast cancer therapy
Source: Smart Med. 2022 Dec 27;1(1):e20220036. doi: 10.1002/SMMD.20220036 (PMC11235718; doi:10.1002/SMMD.20220036)
Supplement: Supplementary file 1 — Supporting Information S1 [file SMMD-1-e20220036-s001.docx]

**Supplemental file**

**Co-delivery CPT and PTX prodrug with a photo/thermo-responsive nanoplatform for triple negative breast cancer therapy**

*Wenhui Zhou, Xiaodong Ma, Jie Wang, Xiaoyu Xu, Oliver Koivisto, Jing Feng, Tapani Viitala, Hongbo Zhang^*^*


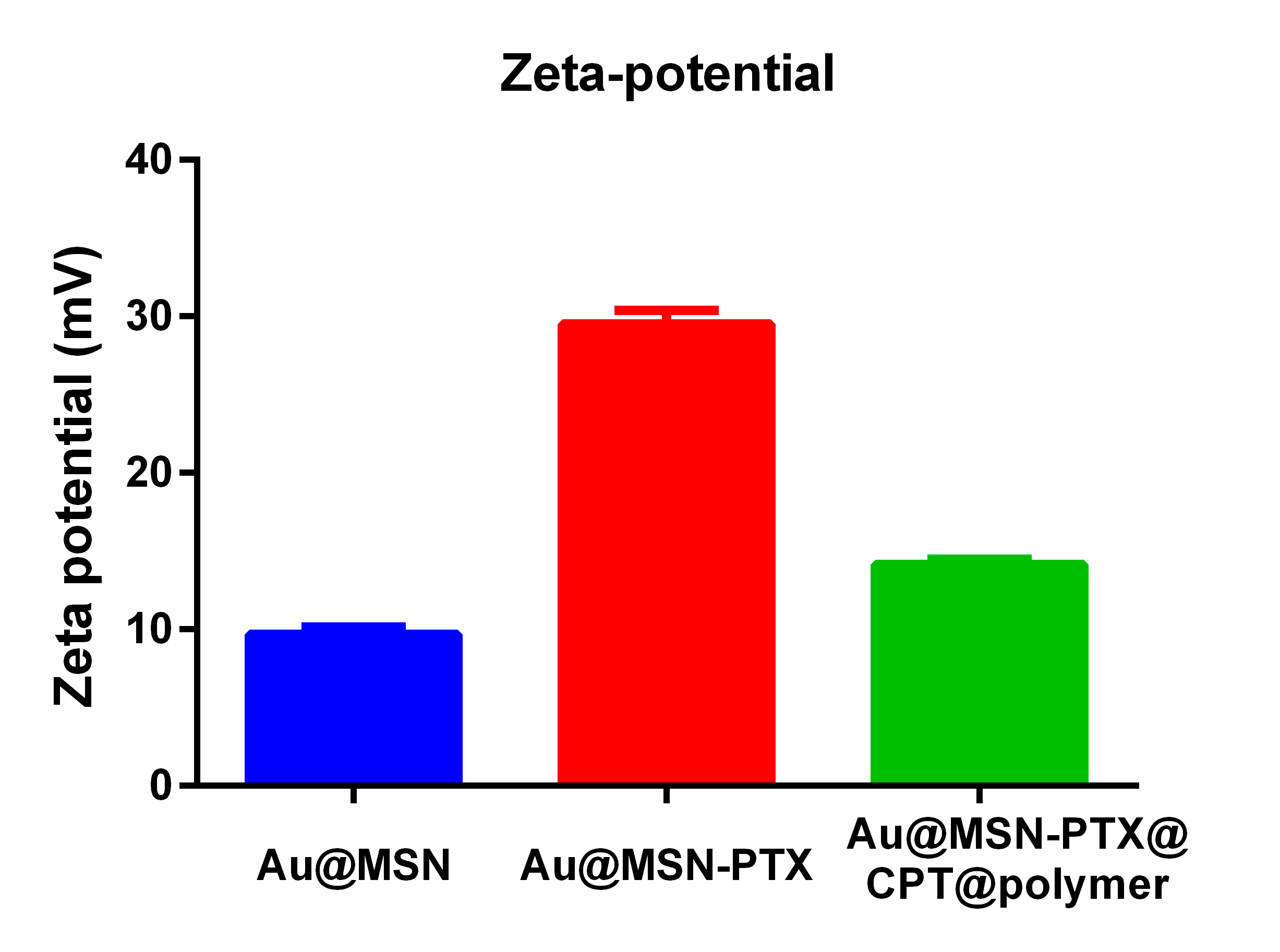


**Figure S1.** Zeta potential of Au@MSN-NH_2_, Au@MSN-PTX and Au@MSN-PTX@CPT@polymer NPs.

**

**

**Figure S2.** The fluorescence intensity of CPT-positive MDA-MB-231 cells after treatment with 10 μg/ml Au@MSN-PTX@CPT@polymer NPs for 2-6 hours.

**
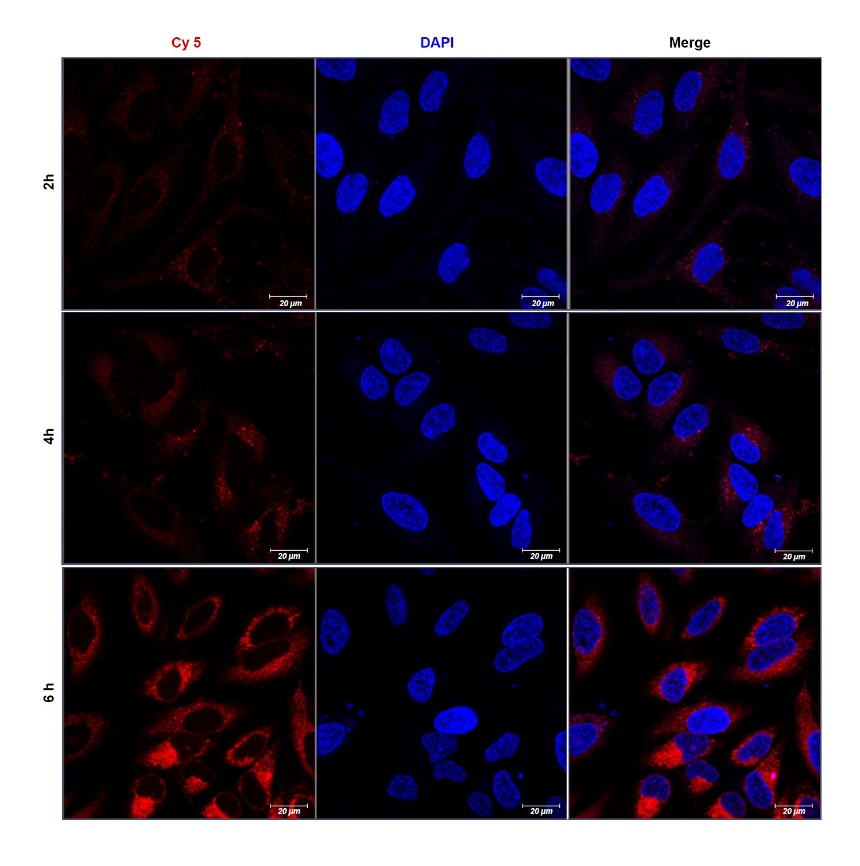
**

**Figure S3.** Representative confocal microscopy images of MDA-MB-231 cells after treatment with 10 μg/ml Cy 5-labeled Au@MSN-PTX@CPT@polymer NPs for 2-6 hours (red: Cy 5; blue: DAPI; scale bar: 20 μm).

**
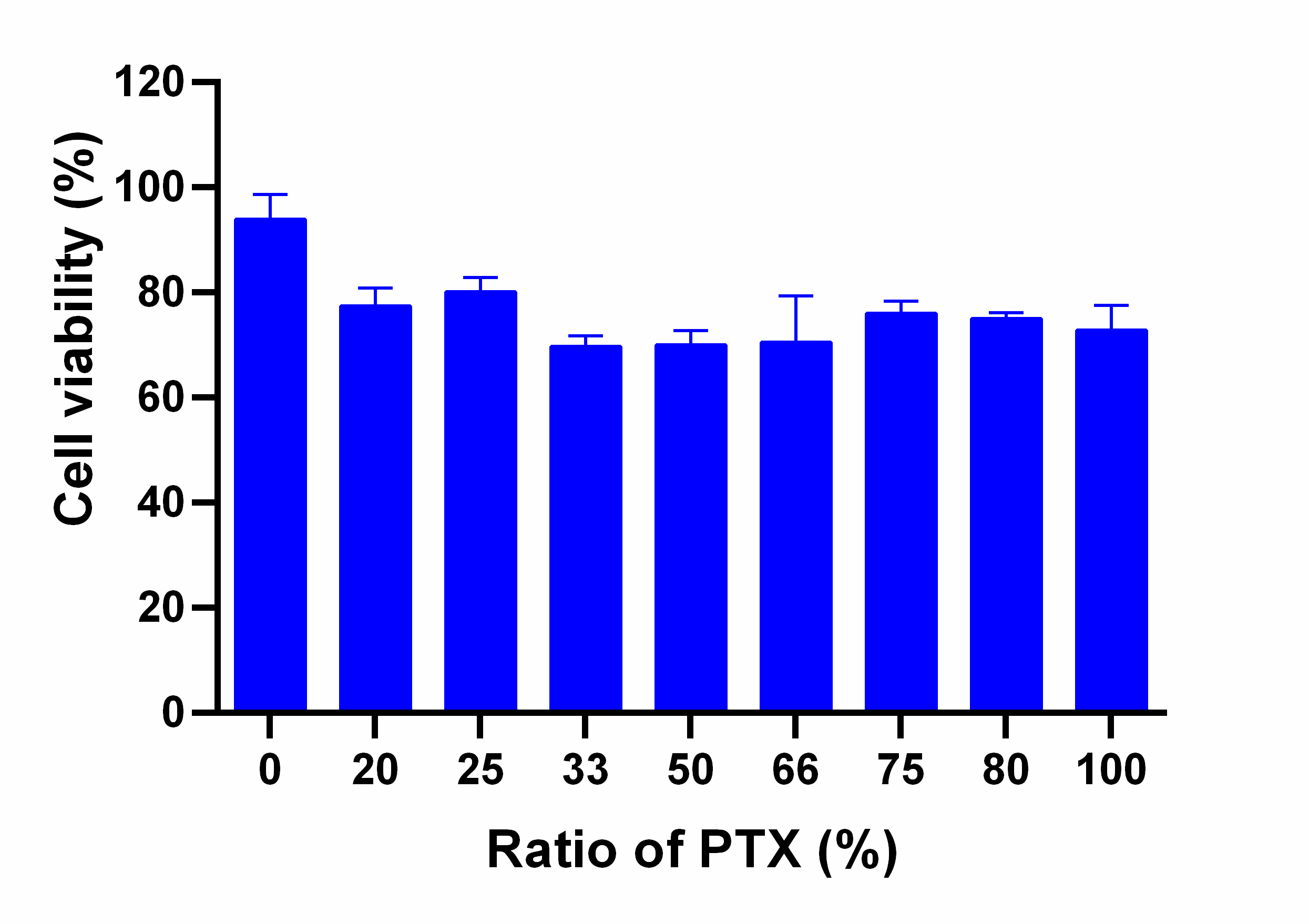
**

**Figure S4.** Cell viability of MDA-MB-231 cells after treatment with PTX + CPT (10 μg/ml in total) for 48 hours.
